# A two-state hysteresis model from high-dimensional friction

**DOI:** 10.1098/rsos.150188

**Published:** 2015-07-29

**Authors:** Saurabh Biswas, Anindya Chatterjee

**Affiliations:** Department of Mechanical Engineering, IIT Kanpur, Kanpur, Uttar Pradesh, India

**Keywords:** hysteresis, minor loops, Iwan model, model reduction, parameter fitting

## Abstract

In prior work (Biswas & Chatterjee 2014 *Proc. R. Soc. A* 470, 20130817 (doi:10.1098/rspa.2013.0817)), we developed a six-state hysteresis model from a high-dimensional frictional system. Here, we use a more intuitively appealing frictional system that resembles one studied earlier by Iwan. The basis functions now have simple analytical description. The number of states required decreases further, from six to the theoretical minimum of two. The number of fitted parameters is reduced by an order of magnitude, to just six. An explicit and faster numerical solution method is developed. Parameter fitting to match different specified hysteresis loops is demonstrated. In summary, a new two-state model of hysteresis is presented that is ready for practical implementation. Essential Matlab code is provided.

## 1. Introduction

In this paper, we follow up on our recent work on low-dimensional modelling of frictional hysteresis [1]. Contributions of this paper include a different underlying frictional model with greater intuitive appeal, new analytical insights, reduction in the number of states from six to two,^1^ reduction in the number of free parameters by an order of magnitude, and demonstration of fitting these parameters to several hypothetical hysteresis loops. The net result is a two-state hysteresis model that captures minor loops under small reversals within larger load paths and is ready for practical numerical implementation (simple Matlab code is provided).

For elementary background, we note that hysteresis is a largely rate-independent, irreversible phenomenon that occurs in many systems. Much research on hysteresis has been done over several decades (e.g. three volumes of Bertotti & Mayergoyz [2] and references therein). For classical papers, see, for example, Ewing [3], Rowett [4], Preisach [5], Jiles & Atherton [6]. For our present purposes, for hysteresis in mechanical systems with elastic storage and frictional dissipation, a model due to Iwan [7,8] seems promising, but is high-dimensional and deeply nonlinear with several dry friction elements. By contrast, the famous Bouc–Wen model ([9,10]; see also [11]) is one-dimensional but fails to form minor loops under small reversals within larger load paths.

With this background, we recently studied [1] a frictional hysteretic system given by

1.1 $$\mu \;\mathrm{sgn}(\dot{x})+Kx=bf(t),$$

where *x* is high-dimensional; *μ* is diagonal; *K* is symmetric and positive definite; *b* is a column matrix; *f*(*t*) is scalar and differentiable; and the *signum* function ‘sgn’ is defined elementwise as follows:

$$\mathrm{sgn}(u)\begin{array}{ll} =+1, & u>0,\\ =-1, & u<0,\\ \in [-1,1], & u=0. \end{array}$$

Equation (1.1) can be solved incrementally via a linear complementarity problem (LCP) [12] or, less efficiently, using other means as described later. The solution of equation (1.1) captures important aspects of hysteresis including formation of minor loops. From equation (1.1), we had developed a reduced order model with six states. The order reduction included finding the slip direction as a minimizer of a complicated function containing many signum nonlinearities, for which a convenient analytical approximation was found. However, some shortcomings remained. The choice of basis vectors involved some arbitrariness whose implications were unclear; reductions below sixth order gave poor results; and there were too many fitted parameters for practical use.

In the light of the above, this paper makes the following notable progress. A more intuitively appealing frictional system is studied here, motivated by the Iwan model [7] and yielding a simpler governing equation. The numerically obtained basis vectors are now amenable to analytical approximation, providing better analytical insight. Finally, a two-state, reduced order model is derived that allows practical parameter fitting to match a range of given data.

As far as we know, the two-state model developed here has no parallel in the literature.

## 2. New frictional system

**Figure 1.**
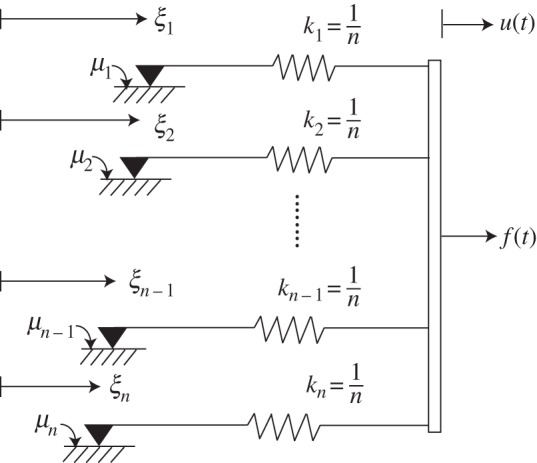
A high-dimensional frictional system.

Differing somewhat from Biswas & Chatterjee [1], here we consider the intuitively simpler high-dimensional frictional system sketched in figure 1. In this *n*-dimensional model (with *n* large), each spring has stiffness 1/*n*, and friction coefficients at the slip sites are

$$\mu_{1}=\frac{\mu_{0}}{n},\;\mu_{2}=\frac{2\mu_{0}}{n},\ldots ,\mu_{n}=\frac{n\mu_{0}}{n}=\mu_{0}.$$

As indicated in the figure, *u*(*t*) is a displacement input to the system, for which a force *f*(*t*) is needed. Friction forces at the slip sites are written as

$$F_{1}=-\mu_{1}\;\mathrm{sgn}({\dot{\xi }}_{1}),\;F_{2}=-\mu_{2}\;\mathrm{sgn}({\dot{\xi }}_{2}),\ldots ,F_{n}=-\mu_{n}\;\mathrm{sgn}({\dot{\xi }}_{n}),$$

where the overdot denotes a time derivative and the signum function is understood to be multivalued at zero (taking any necessary value between ±1). The governing equation is

$$\mu_{j}\;\mathrm{sgn}({\dot{\xi }}_{j})+\frac{1}{n}\xi_{j}=\frac{1}{n}u(t),\;j=1,\ldots ,n$$

In matrix form

2.1 $$\mu \;\mathrm{sgn}(\dot{\xi })+K\xi =bu(t),$$

which resembles equation (1.1) but is in fact simpler because the elements of *μ* now have a regular variation (they are linearly increasing), *K* is a scalar multiple of the identity matrix, and all elements of *b* are identical. The output force *f*(*t*) is

2.2 $$f(t)=\sum_{j=1}^{n}k_{j}(u(t)-\xi_{j})=u(t)-\frac{1}{n}\sum_{j=1}^{n}\xi_{j}.$$

Incidentally, if a spring of stiffness *k*_s_ is attached to the system, in parallel, being stretched by *u*(*t*), then the net output force is

2.3 $$f(t)=(1+k_{s})u(t)-\frac{1}{n}\sum_{j=1}^{n}\xi_{j}.$$

We will use the parameter *k*_s_ later for better fitting of the model to specified hysteretic response curves; here, we note that *k*_s_ in equation (2.3) has no effect on the solution of equation (2.1), which takes *u*(*t*) as its input.

We solve equation (2.1) incrementally by casting it first into an LCP (as described in Biswas & Chatterjee [1]) and then by using Lemke's algorithm (as implemented by Miranda & Fackler [13]). There is in fact a large literature on solving friction problems using the LCP; readers interested in the theory may consult, e.g. Klarbring & Pang [14].

Alternatively, equation (2.1) may be regularized as follows:

2.4 $$\dot{\xi }=\mathrm{sgn}(bu-K\xi )\cdot \exp\left(\frac{|\mu^{-1}(b\;u-K\xi )|-1}{\epsilon }\right),\;0<\epsilon \ll 1.$$

In the above the exponential, the absolute value within it, and the signum function are all evaluated elementwise; the fact that *K* is a scalar multiple of the identity and that *μ* is diagonal has been used to simplify the first term; and *ϵ* is a regularizing parameter. The justification for this regularizing method is that (i) the exponential term produces high rates of change only when the concerned absolute value exceeds unity, and (ii) the signum term outside guides that rate of change in the correct direction. Further discussion of this regularizing method is avoided to minimize the distraction from the main flow of the paper, but a numerical example is given in appendix A. Note that equation (2.4) may be solved using an ordinary differential equation (ODE) solver.

For our numerical solution, the arbitrarily selected numerical parameters are as follows. We use *n*=500, *μ*_0_=0.002 and the two-frequency displacement input

2.5 u(t)=0.6748sin⁡(t)+0.2887sin⁡(6.5581t).

We then solve equation (2.1) incrementally using 12 000 uniform time steps of d*t*=0.001 each.

**Figure 2.**
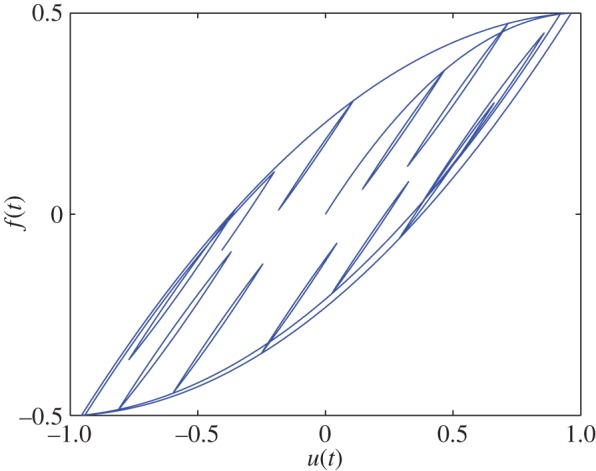
Hysteresis curve obtained for the 500 dimensional frictional system with *u*(*t*) as in equation (2.5). Note the minor loops, as mentioned in §1.

In this way, we obtain a 12 000×500 matrix, wherein each row is *ξ*^T^ at some instant. From *ξ*, we find *f*(*t*) using equation (2.2). Figure 2 shows *f*(*t*) against *u*(*t*). Minor loops are seen. As emphasized by Biswas & Chatterjee [1], such minor loops are not predicted by the Bouc–Wen model or indeed any hysteresis model with a single state.

We now develop a reduced-order model from this high-dimensional hysteretic system (figure 1, equation (2.1)).

## 3. Reduced-order model

Our system is discrete. However, the largeness of *n* and the slow variation in *μ*_*j*_ suggest that we might loosely think of an approximating continuous system. With this motivation, we assume

3.1 $$\xi_{j}\approx \sum_{r=1}^{m}q_{r}(t)\phi_{r}(x_{j}),\;x_{1}=1,x_{2}=2,\ldots .$$

In the above, *m* is the reduced dimension, *q*_*r*_(*t*)'s are functions of time and the *ϕ*_*r*_(*x*)'s are basis functions yet to be chosen.

### 3.1 Choice of basis functions

**Figure 3.**
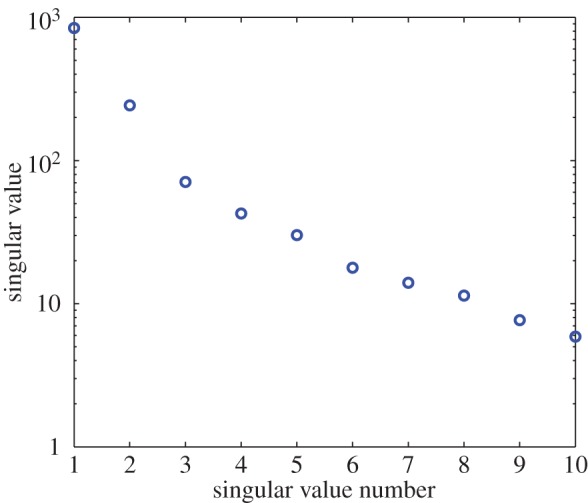
First 10 singular values of *ξ*.

**Figure 4.**
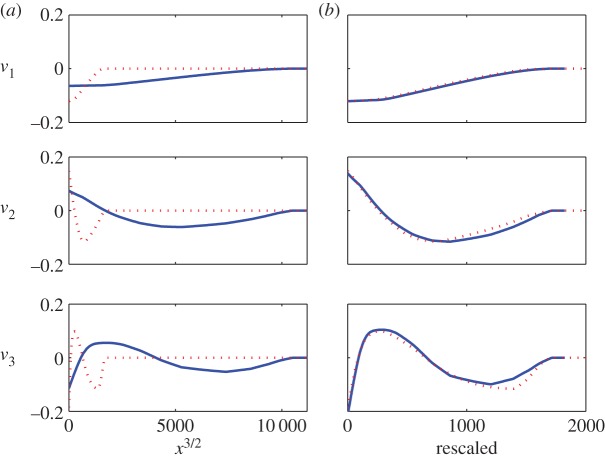
(*a*) First three singular vectors plotted against *x*^3/2^. The blue solid curves are solutions for *μ*_0_=0.002 and *u*(*t*) as in equation (2.5); the red dotted curves are solutions for *μ*_0_=0.004 and the different, also arbitrary, u(t)=0.4049sin⁡(t)+0.1732sin⁡(4.5581t). (*b*) Singular vectors for the two cases (*a*) match fairly well after scaling horizontally and vertically.

The singular value decomposition^2^ of the 12 000×500 data matrix from §2 shows that the first two singular values are distinctly larger than the rest (figure 3). Figure 4*a* shows the first three singular vectors plotted against *x*^3/2^ (where *x*=1,2,…,500) for two different solutions using different *μ*_0_'s and *u*(*t*)'s. Figure 4*b* shows that, after rescaling, the singular vectors for the two cases are similar. These observations suggest that the basis functions may be taken as functions of *x*^3/2^ (the $\frac{3}{2}$ power is empirical, based on the fact that the slope near *x*^3/2^ is finite and non-zero). In order to ensure eventual decay to zero, we choose the following basis functions for lower order modelling:

3.2 $$\phi_{r}(x)=\exp(-\alpha x^{3/2})\cdot {\left(x^{3/2}\right)}^{r-1},\;r=1,\ldots ,m.$$

The free parameter *α*>0 controls the decay rate of the basis functions. The actual discrete versions of these basis functions will be orthonormalized below for analytical convenience.

It may be noted that the ad hoc form of equation (3.2) leads to simplification below, but also means that the possibility of a very good match with the full numerical solution has now been abandoned.

### 3.2 Slip criterion

Our *slip criterion* is this: slip cannot occur if the accompanying frictional dissipation exceeds the external work input minus the internal increase in potential energy. This criterion will help us find slip directions and rates below.

What follows in §3.2–3.4 has much in common with Biswas & Chatterjee [1], but is included for completeness. Let

ξ≈Φq

where the columns of *Φ* are basis vectors from equation (3.2), but orthonormalized so that *Φ*^T^*Φ*=*I*. Then the slip rate vector

$$v\approx \Phi \dot{q}=\Phi \eta \text{ (say)}$$

with

$$v^{T}v=\eta^{T}\Phi^{T}\Phi \eta =\eta^{T}\eta .$$

The rate of frictional dissipation is

3.3 $$\sum_{j=1}^{n}|{\dot{\xi }}_{j}|\mu_{j}=\sum_{j=1}^{n}{\dot{\xi }}_{j}\mu_{j}\;\mathrm{sgn}({\dot{\xi }}_{j})=\eta^{T}\Phi^{T}\mu \;\mathrm{sgn}(\Phi \eta ).$$

The rate of increase in spring potential energy is

3.4 $$\frac{d}{dt}\left(\sum_{j=1}^{n}\frac{1}{2n}{\left(u-\xi_{j}\right)}^{2}\right)=\frac{1}{n}\sum_{j=1}^{n}\left(\dot{u}-{\dot{\xi }}_{j})(u-\xi_{j}\right)$$

and the rate of work done by the external force *f* is

3.5 $$\dot{u}f=\dot{u}\sum_{j=1}^{n}\frac{1}{n}(u-\xi_{j}).$$

Substituting the above into our slip criterion yields

3.6 $$\eta^{T}\Phi^{T}\mu \;\mathrm{sgn}(\Phi \eta )+\eta^{T}(\Phi^{T}K\Phi )q-\eta^{T}(\Phi^{T}b)u\leq 0,$$

with matrices *μ*, *K* and *b* as described in §2. We define

3.7 $$\eta^{T}\Phi^{T}\mu \;\mathrm{sgn}(\Phi \eta )=G(\eta )$$

and

3.8 $$(\Phi^{T}K\Phi )q-(\Phi^{T}b)u=\bar{K}q-\bar{b}u=c,$$

so that inequality (3.6) becomes

3.9 $$G(\eta )+\eta^{T}c\leq 0.$$

We note that $\bar{K}=(1/n)I$ above, with *I* being the identity matrix; and choosing *n*=500,

$$\bar{b}=\left\{\begin{array}{c} 0.0326\\ 0.0068 \end{array}\right\}\;\text{and}\;\left\{\begin{array}{c} 0.0285\\ 0.0059 \end{array}\right\}$$

for *α*=0.0008 and *α*=0.0012, respectively. These values will be used later.

If the *minimum* possible value of the left-hand side of inequality (3.9) is negative, rapid slip will occur because there is no inertia; if the minimum is positive, no slip can occur; and if it is zero over a time interval, sustained slip can occur at a finite rate.

Accordingly, we will minimize *G*(*η*)+*η*^T^*c* at each time step with respect to *η*, and see if the minimum is negative or positive. Noting that *G*(*η*)+*η*^T^*c* is homogeneous of degree one in *η*, our minimization will be done subject to *η*^T^*η*=1. The only difficulty is that *G*(*η*) is a complicated function. Luckily, a convenient analytical approximation for *G*(*η*) of equation (3.7) was found by Biswas & Chatterjee [1].

### 3.3 Approximation of *G*(*η*)

*G*(*η*) is homogeneous of degree one in *η*. In Biswas & Chatterjee [1], a similar function was encountered and the following approximation was considered:

3.10 $$G(\eta )\approx \frac{{\left(\eta^{T}A\eta \right)}^{\beta }}{{\left(\eta^{T}\eta \right)}^{\beta -0.5}},$$

with *A* a fitted symmetric and positive definite matrix; also, *β*=0.5 was found to be near-optimal and selected due to analytical convenience. We use the same approximation here (again with *β*=0.5).

Fitting of the matrix *A* was described in detail in the earlier paper. Here we fix *n*=500, let *μ*_0_ vary, and fit *A* for *α*=0.0008 and *α*=0.0012. We find that to an excellent approximation

3.11 $$A=\mu_{0}^{2}\bar{A}$$

with

$$\bar{A}=\left[\begin{array}{cc} 24.8170 & -10.0836\\ -10.0836 & 6.2967 \end{array}\right]$$

and

$$\bar{A}=\left[\begin{array}{cc} 11.1372 & -4.5175\\ -4.5175 & 2.8222 \end{array}\right]$$

for *α*=0.0008 and *α*=0.0012, respectively. With *A* as above and *β*=0.5, *G*(*η*) has been approximated; we now turn to the slip direction.

### 3.4 Slip direction

The slip direction *η* minimizes *y*=*G*(*η*)+*η*^T^*c* for given

$$c=\frac{1}{n}q-\bar{b}u,$$

subject to *η*^T^*η*=1.

Introducing a Lagrange multiplier for the constraint, using the approximation $G(\eta )\approx \sqrt{\eta^{T}A\eta }$, taking the gradient with respect to *η*, and letting $2\lambda =\bar{\lambda }$, we obtain

3.12 $$\frac{1}{\sqrt{\eta^{T}A\eta }}A\eta -\bar{\lambda }\eta =-c.$$

The minimizing 2×1 matrix *η* is found by solving a 4×4 eigenvalue problem (see appendix B). If the corresponding

3.13 $$y=\sqrt{\eta^{T}A\eta }+\eta^{T}c<0,$$

then slip occurs in the direction of *η*.

### 3.5 Reduced-order model using incremental map

The unit vector *η* (computed as outlined in appendix B) gives the direction of slip, but the actual rate of slip $\dot{q}$ remains to be found. In Biswas & Chatterjee [1], we used a stiff system of ODEs with a large *ad hoc* gain parameter. Here, we use a faster explicit incremental formulation as follows.

During time increment Δ*t*, let Δ*q*=*η*Δ*s* for some Δ*s*≥0. Holding *η* fixed during the time increment, we find from equations (3.13) and (3.8)

3.14 $$\Delta y=\eta^{T}\Delta c=\eta^{T}(\bar{K}\Delta q-\bar{b}\Delta u).$$

It follows that

3.15 $$y+\Delta y=\sqrt{\eta^{T}A\eta }+\eta^{T}c+\eta^{T}(\bar{K}\eta \Delta s-\bar{b}\Delta u).$$

Equation (3.15) gives a linear relationship between the unknowns Δ*s* and *y*+Δ*y* (with all other things including Δ*u* being known). The unknowns satisfy the linear complementarity conditions

3.16 Δs≥0,y+Δy≥0,Δs⋅(y+Δy)=0.

Solving^3^ equations (3.15) and (3.16) yields Δ*s*, which in turn gives the increment in *q* through

3.17 q(t+Δt)=q(t)+ηΔs.

The output force *f*(*t*), from equations (2.2), (2.3) and (3.1), is

3.18 $$f(t)=(1+k_{s})u(t)-q^{T}\bar{b}.$$

**Figure 5.**
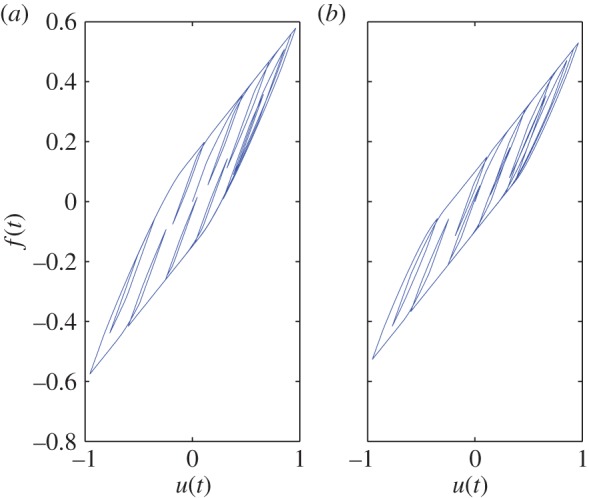
Hysteresis curves for *u*(*t*) as in equation (2.5). (*a*) *α*=0.0008 and (*b*) *α*=0.0012.

Figure 5 shows results for *u*(*t*) as in equation (2.5), and with *k*_s_=0. We used *n*=500, *μ*_0_=0.002, and *α*=0.0008 and 0.0012. Both solutions capture minor loops. The hysteresis loops obtained depend on the parameter *α*.

As mentioned earlier, a direct comparison of these results with the earlier high-dimensional simulation is not meaningful because we have adopted analytical expressions for the basis functions (equation (3.2)) instead of numerically computed proper orthogonal modes from actual solutions (figure 4). The high-dimensional model has thus motivated the structure of the lower dimensional model, and now we work directly with the latter.

### 3.6 Final reduced-order model using a differential equation

Although the numerical results that follow were obtained using the incremental map given above, some users may prefer to have a differential equation for the hysteresis model. We now present one. We also present the entire hysteresis model compactly and algorithmically below. A detailed example calculation is given in appendix C.

The quantities assumed given are:

1. System matrices. These are:
  (i) An *m*×*m* symmetric and positive definite matrix *A* (we have been working with *m*=2). Since *A* can be diagonalized by an orthogonal coordinate transformation, for compactness we henceforth assume that *A* is diagonal, with elements increasing order. In the *m*=2 case, we introduce a scalar factor $\bar{\mu }>0$ to write
    $$A=\bar{\mu }\left[\begin{array}{cc} \sigma & 0\\ 0 & 1 \end{array}\right],\;0<\sigma <1.$$
  (ii) A matrix $\bar{K}$, which is a scalar multiple of the identity. Here
    $$\bar{K}=\bar{\alpha }\left[\begin{array}{cc} 1 & 0\\ 0 & 1 \end{array}\right],\;\bar{\alpha }>0.$$
  (iii) An *m*×1 column matrix $\bar{b}$. Here
    $$\bar{b}=\left\{\begin{array}{c} {\bar{b}}_{1}\\ {\bar{b}}_{2} \end{array}\right\}.$$
2. System inputs: *u* is the system input, and $\dot{u}$ is known at each instant.
3. The state vector: *q* is an *m*×1 state vector (here *m*=2).

Given the above system matrices and inputs, we first compute $c=\bar{K}q-\bar{b}u$. Subsequently, the possible slip direction *η* is computed as a function of *A* and *c* as described in appendix B, using straightforward matrix calculations of order 2*m*.

Using the above, we define the intermediate quantity^4^

3.19 $$\dot{s}=\left[\frac{\eta^{T}\bar{b}}{\eta^{T}\bar{K}\eta }\dot{u}-My|\dot{u}|\right]\cdot \{y\leq 0\},$$

where *M* is a user-defined positive number (we have used *M*=1 with good results); and where {*y*≤0} is a logical variable (1 if the condition holds, and 0 otherwise).

Finally, recalling equation (3.16), we write

3.20 $$\dot{q}=\eta \dot{s}\cdot \{\dot{s}>0\},$$

where $\left\{\dot{s}>0\right\}$ is a logical variable as above (1 if the condition holds, and 0 otherwise). At this point, starting from the state matrices, the inputs *u* and $\dot{u}$, and the state vector *q*, we have computed $\dot{q}$.

The above system of ODEs does not need a large ad hoc ‘gain’ parameter as was used in Biswas & Chatterjee [1].

## 4. Fitting parameters to given data

As described in §3.5, for a two-state model we have five fitted parameters ($\bar{\mu }$, *σ*, $\bar{\alpha }$, ${\bar{b}}_{1}$ and ${\bar{b}}_{2}$). If we used a three-state model, we would add one diagonal entry in *A*, no new parameters to $\bar{K}$, and one element to $\bar{b}$, obtaining a model with seven fitted parameters.

Introduction of an added spring in parallel with constant *k*_s_, as in equation (2.3), would make it six fitted parameters for the two-state model and eight fitted parameters for the three-state model. For clarifying that a spring in parallel is implied, we will refer to these latter two as 5+1 and 7+1, respectively.

**Figure 6.**
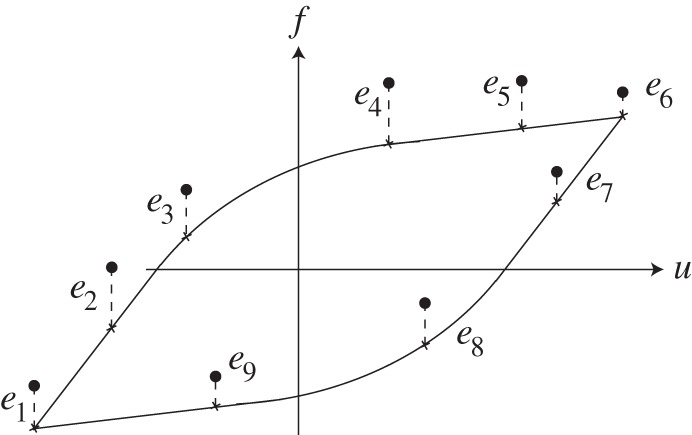
Fitting a hysteresis curve. Dotted points represent given data. Vertical distances between data and fitted curve are squared and added. That sum is minimized with respect to the fitted parameters.

We now fit some hypothetical hysteresis loops using our two state, 5+1 parameter model, using nonlinear least squares as depicted schematically in figure 6 (the minimization was done using Matlab's built-in function fminsearch).

## 5. Results and discussion

We now show results of fitting the hysteresis model to some arbitrary input data. Results are depicted graphically here; numerical values of fitted parameters are given in appendix D.

**Figure 7.**
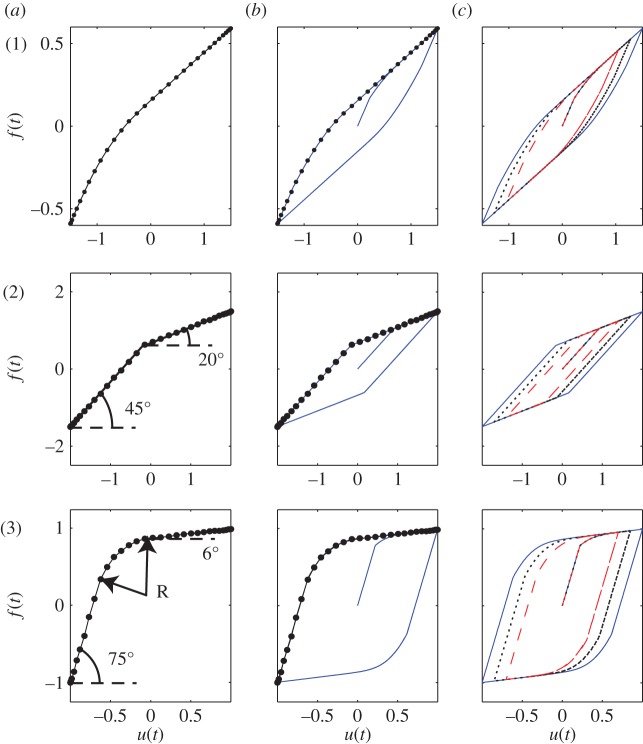
Fitting of hysteresis loops. Part (*a*): prescribed hysteretic shape (only half of the cycle). Part (*b*) corresponding fitted hysteresis loop. Part (*c*) fitted hysteresis loop (blue solid) along with two hysteresis loops corresponding to 85% (black dotted) and 70% (red dashed) of the amplitude.

Figure 7 shows fitting of three hysteresis loops, numbered 1 to 3. Each case is depicted in three parts, namely (*a*), (*b*) and (*c*). Parts 1(*a*) to 3(*a*) show the prescribed or desired loop shapes (half the cycle). Parts 1(*b*) to 3(*b*) show the corresponding hysteresis loops obtained by fitting parameters. These fitted parameters are then used to plot hysteresis loops using smaller (85 and 70%) input amplitudes, and parts 1(*c*) to 3(*c*) show these loops corresponding to 100% (blue solid), 85% (black dotted) and 70% (red dashed) amplitude.

**Figure 8.**
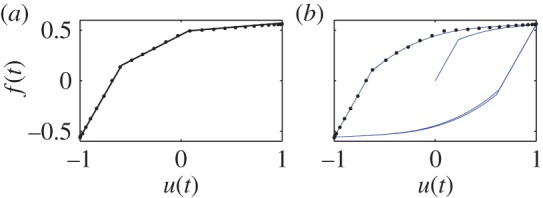
(*a*) Given data, made of three straight lines. (*b*) Corresponding fitted hysteresis loop. The model apparently cannot capture two sharp slope changes.

The model fits the above given data (figure 7) well. However, the model does not work well for hysteresis curves with two distinct changes of slope. For example, figure 8 shows how a hysteresis curve made of three straight lines is not captured very accurately by the two-state model (or even, in attempts not documented here, by models with three or four states). However, overall, our model fits a reasonable range of data usefully well.

**Figure 9.**
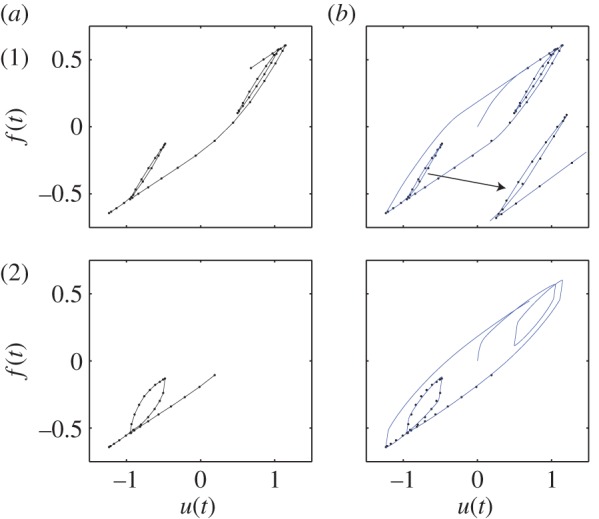
(*a*) Given data for hysteresis loops with minor loops. (*b*) Corresponding fitted hysteresis loops. Case 1(*a*): two minor loops are specified within the major loop. Case 2(*a*): thicker minor loop than in Case 1(*a*). 1(*b*) and 2(*b*): nature of complete loops.

In figure 9, we consider fitting of minor loops. In case 1(*a*), we specify two minor loops within a major loop. The corresponding fit is shown in 1(*b*). In case 2(*a*), we try to thicken one of the minor loops of case 1(*a*). The model captures the loop thickening in the third quadrant of the plot, but similar changes occur in the first quadrant as well, because our hysteresis loops are symmetrical about the origin.

The model developed in this paper, as explained and demonstrated above, has several advantages over our earlier work [1]. These advantages include a more intuitive underlying frictional system, analytical insights into basis functions, a minimal number of states (two), a small number of fitted parameters, and the ability to match a reasonable range of hysteretic behaviours.

The computational complexity of our model exceeds that of the Bouc–Wen model but compensates by capturing minor loops. Direct computation with the Iwan model for arbitrary forcing, in high dimensions, would be significantly more complex than for our model.

Further study may clarify the precise advantages of including more than two states in the hysteresis model; why (apparently) two distinct changes in slope are difficult for the model to capture; and how parameter fitting can be done more reliably and efficiently than using general purpose minimization routines with random initial guesses.

New research questions might also now be addressed somewhat more easily; these include control strategies for such hysteretic systems, as well as the nonlinear dynamics of systems that include elements with such hysteretic behaviour.

## 6. Closing note

An anonymous reviewer of this article has brought to our attention the recent work by Scerrato *et al.* [16,17]. These works are interesting and complementary to our approach, possibly opening up new lines of research for both.

In particular, in these works, a micro-structural model for dissipation in concrete is considered, while our starting model is abstract although physical, and not motivated by any specific material. Both models allow complex stress histories, but their work considers multiaxial stress states while ours so far does not. In that work, the experimental hysteresis loops fitted are asymmetric, while ours are symmetric. We have fewer state variables than they do.

Thus, their work motivates our approach to try to incorporate triaxiality in stress and asymmetry in hysteresis loops, while our work suggests new methods of approximation and model-order reduction that may lead to improvements in their approach.

## Figures and Tables

**Figure 10.**
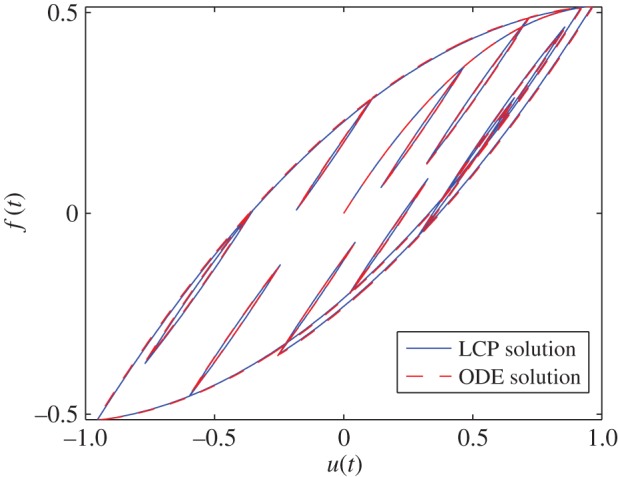
Hysteresis curves obtained for a 50 dimensional system from LCP and ODE solutions.

**Figure 11.**
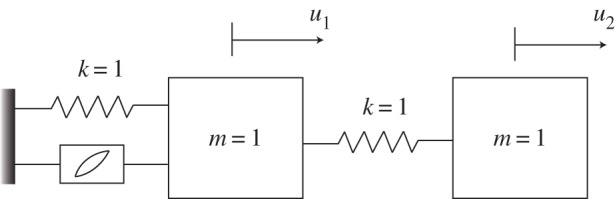
A two degree of freedom spring–mass system with a hysteretic damper.

**Figure 12.**
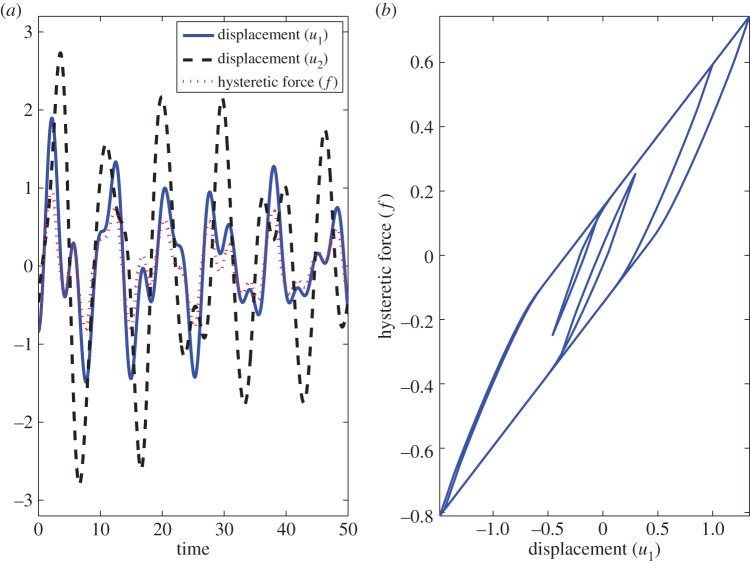
(*a*) Displacements *u*_1_, *u*_2_ and hysteretic force *f* versus time. (*b*) Hysteresis between *u*_1_ and *f* for a portion of the computed solution.

## Footnotes

1 Two is the theoretical minimum number. Single-state models cannot capture commonly observed behaviour (see fig. 3 in [1]).

2 Also known as the proper orthogonal decomposition (e.g. [15]).

3 Since Δ*s* and *y* are scalars, these equations can be solved easily, and LCP code is not needed.

4 The way $\dot{s}$ is defined ensures that if *y*>0, then $\dot{s}=0$; and otherwise, the first term inside the square brackets maintains $\dot{y}=0$ (see equation (3.15)) while the second term drives *y* from any negative values it takes during simulation towards zero. Since the −*My* term is a small stabilizing correction, *M* does not need to be very large.

## Appendix A. Linear complementarity problem and ordinary differential equation solutions compared

An anonymous reviewer of an earlier version of this paper asked about the possibility of other solutions than what the LCP predicts. It is true that such high-dimensional frictional systems can have non-unique solutions. The aim of our paper has been to *derive* a low-dimensional hysteresis model rather than probe the full complexities of the original high-dimensional model. Nevertheless, here we demonstrate that the LCP solution is close to the solution obtained from a system of ODEs (namely equation (2.4)) obtained by regularizing the frictional forces in the model.

Figure 10 shows a comparison between LCP and ODE results from equations (2.1) and (2.4). Here, we have used *n*=50, *μ*_0_=0.02, and *u*(*t*) as in equation (2.5). We also chose *ϵ*=0.003 in equation (2.4). Both the solutions match quite well. The LCP solution is much faster and therefore adopted for larger *n*.

## Appendix B

**B.1. Finding the slip direction *η***

We have been able to substantially simplify the calculation of the slip direction *η*, compared to our original method developed by Biswas & Chatterjee [1]. Recall §3.5, where it was explained that using an orthogonal transformation we can take *A* to be diagonal:

$$A=\bar{\mu }\left[\begin{array}{cc} \sigma & 0\\ 0 & 1 \end{array}\right].$$

We reproduce equation (3.12) below:

$$\frac{A}{\sqrt{\eta^{T}A\eta }}\eta -\bar{\lambda }\eta +c=0.$$

Let $\bar{\lambda }=\left(1/\sqrt{\eta^{T}A\eta }\right)\hat{\lambda }$. Then,

B 1 $$\frac{1}{\sqrt{\eta^{T}A\eta }}(A-\hat{\lambda }I)\eta =-c$$

or

B 2 $$\frac{1}{\sqrt{\eta^{T}A\eta }}\left(\bar{\mu }\left[\begin{array}{cc} \sigma & 0\\ 0 & 1 \end{array}\right]-\hat{\lambda }\left[\begin{array}{cc} 1 & 0\\ 0 & 1 \end{array}\right]\right)\left\{\begin{array}{c} \eta_{1}\\ \eta_{2} \end{array}\right\}=-\left\{\begin{array}{c} c_{1}\\ c_{2} \end{array}\right\}.$$

From equation (B 2)

B 3 $$\eta_{1}=-\frac{c_{1}\sqrt{\eta^{T}A\eta }}{\left(\bar{\mu }\sigma -\hat{\lambda }\right)}$$

and

B 4 $$\eta_{2}=-\frac{c_{2}\sqrt{\eta^{T}A\eta }}{\left(\bar{\mu }-\hat{\lambda }\right)}.$$

Since $\eta^{T}A\eta =\bar{\mu }(\eta_{1}^{2}\sigma +\eta_{2}^{2})$, by equations (B 3) and (B 4), we have

$$\bar{\mu }\left(\frac{c_{1}^{2}\sigma }{{\left(\bar{\mu }\sigma -\hat{\lambda }\right)}^{2}}+\frac{c_{2}^{2}}{{\left(\bar{\mu }-\hat{\lambda }\right)}^{2}}\right)=1,$$

which yields

B 5 $${\left(\bar{\mu }\sigma -\hat{\lambda }\right)}^{2}{\left(\bar{\mu }-\hat{\lambda }\right)}^{2}-\bar{\mu }{\left(\bar{\mu }-\hat{\lambda }\right)}^{2}c_{1}^{2}\sigma -\bar{\mu }{\left(\bar{\mu }\sigma -\hat{\lambda }\right)}^{2}c_{2}^{2}=0.$$

Now, somewhat fortuitously, we consider the 4×4 matrix

B 6 $$B=\left[\begin{array}{cc} A & cc^{T}\\ A & A \end{array}\right].$$

It turns out that $\hat{\lambda }$ of equation (B 5) is an eigenvalue of the above *B*. Setting

$$\det\left([\begin{array}{cc} A-\hat{\lambda }I & cc^{T}\\ A & A-\hat{\lambda }I \end{array}]\right)=0,$$

we obtain

B 7 $$\det({\left(A-\hat{\lambda }I\right)}^{2}-Acc^{T})=0.$$

We now use a matrix determinant lemma (e.g. [18]) which says that for a square matrix *H* and column matrices *g* and *h* of appropriate sizes,

$$\det(H+gh^{T})=\det(H)(1+h^{T}H^{-1}g).$$

By this lemma, equation (B 7) gives

$$\det{\left(A-\hat{\lambda }I\right)}^{2}\cdot (1-c^{T}{\left[{\left(A-\hat{\lambda }I\right)}^{2}\right]}^{-1}Ac)=0$$

or

B 8 $${\left(\bar{\mu }\sigma -\hat{\lambda }\right)}^{2}{\left(\bar{\mu }-\hat{\lambda }\right)}^{2}\cdot \left(1-\frac{\bar{\mu }\sigma c_{1}^{2}}{{\left(\bar{\mu }\sigma -\hat{\lambda }\right)}^{2}}-\frac{\bar{\mu }c_{2}^{2}}{{\left(\bar{\mu }-\hat{\lambda }\right)}^{2}}\right)=0$$

which, upon multiplying the terms out, gives equation (B 5). Thus, $\hat{\lambda }$ is an eigenvalue of *B*.

Now consider the corresponding eigenvectors

B 9 $$\left[\begin{array}{cc} A-\hat{\lambda }I & cc^{T}\\ A & A-\hat{\lambda }I \end{array}\right]\left\{\begin{array}{c} \psi \\ \zeta \end{array}\right\}=\left\{\begin{array}{c} 0\\ 0 \end{array}\right\},$$

where *ψ* and *ζ* are 2×1. From equation (B 9),

B 10 $$(A-\hat{\lambda }I)\psi +cc^{T}\zeta =0$$

and

B 11 $$A\psi +(A-\hat{\lambda }I)\zeta =0.$$

Premultiplying equation (B 10) with *ζ*^T^ gives

B 12 $$\zeta^{T}(A-\hat{\lambda }I)\psi +{\left(c^{T}\zeta \right)}^{2}=0,$$

while premultiplying equation (B 11) with *ψ*^T^ and then transposing gives

B 13 $$\psi^{T}A\psi +\zeta^{T}(A-\hat{\lambda }I)\psi =0.$$

From equations (B 12) and (B 13), we obtain

B 14 $$c^{T}\zeta =\pm \sqrt{\psi^{T}A\psi }.$$

Dividing equation (B 10) by the scalar quantity *c*^T^*ζ* and then substituting equation (B 14), we obtain

B 15 $$\frac{1}{\sqrt{\psi^{T}A\psi }}(A-\hat{\lambda }I)(\pm \psi )=-c.$$

Since *ψ* is part of an eigenvector it can be scaled such that *ψ*^T^*ψ*=1, and the above equation remains unaffected. However, *ψ* remains indeterminate up to a ‘±’ sign. Comparing equations (B 15) and (B 1), we find *η*=±*ψ*.

Thus, the algorithm for computing *η* is as follows. First, we construct matrix *B* as in equation (B 6). We find its eigenvalues $\hat{\lambda }$. For every real $\hat{\lambda }$, we find the corresponding portion of its eigenvector, *ψ*, normalized to *ψ*^T^*ψ*=1, and with sign chosen such that *ψ*^T^*c*≤0. Searching through all such *ψ* (numbering at least 2 as shown by Biswas & Chatterjee [1], and at most 2*m* which is the size of *B*), we select the one that minimizes $\sqrt{\psi^{T}A\psi }+\psi^{T}c$. That *ψ* is the slip direction *η*.

**B.2. Matlab code for computing *η***

The Matlab code below finds the slip direction *η* given a diagonal *A* and 2×1 vector *c*.

## Appendix C. A two-mass system with a hysteretic damper

Figure 11 shows a two degree of freedom oscillator. There are two unit masses attached by springs of unit stiffness as shown in the figure. A two-state hysteretic damper is attached to the first mass. The displacements of the masses are taken as *u*_1_ and *u*_2_, with *u*_1_ being the input to the hysteretic damper.

We arbitrarily choose the parameter values

$$A=0.117\times 10^{-3}\left[\begin{array}{cc} 0.0638 & 0\\ 0 & 1 \end{array}\right],\;\bar{K}=\frac{1}{500}\left[\begin{array}{cc} 1 & 0\\ 0 & 1 \end{array}\right]\;\text{and}\;\bar{b}=\left\{\begin{array}{c} 0.0326\\ 0.0068 \end{array}\right\}.$$

Equations of motion of the two masses are

C 1 $$\begin{array}{rl} {\ddot{u}}_{1}+2\;u_{1}-u_{2}+f & =0\\ \;\mathrm{and}\;\;{\ddot{u}}_{2}-u_{1}+u_{2} & =0, \end{array}\}$$

where *f* is the hysteretic damper force. In addition, there are two first-order differential equations for the two-dimensional state *q*, as given earlier in §3.5. The hysteretic force *f* is calculated at each instant following equation (3.18):

$$f=u_{1}-q^{T}\bar{b}.$$

Figure 12*a* shows the system response for an arbitrary initial condition. The blue solid line is displacement *u*_1_, the black dashed line is the displacement *u*_2_ and the red dotted line is the hysteretic force *f*. For this two degree of freedom system, the response has more than one frequency. Consequently, *u*_1_ shows short reversals within larger oscillations. Consequently, the hysteretic force shows minor reversals (figure 12*b*).

## Appendix D. Fitted parameters for §5

Numerical values of the fitted parameters for §5 are given in tables 1 and 2.

**Table 1.** Fitted parameters for the cases of figure 7.

case	$\bar{\mu }$	*σ*	$\bar{\alpha }$	${\bar{b}}_{1}$	${\bar{b}}_{2}$	*k*_s_
1	0.0176	0.1425	0.2222	0.2047	0.2458	−0.2481
2	0.1164	0.0047	0.1879	0.0175	0.3734	0.1515
3	0.1506	2.9925	1.4728	−1.7047	−1.4578	2.5444

**Table 2.** Fitted parameters for the cases of figure 9.

case	$\bar{\mu }$	*σ*	$\bar{\alpha }$	${\bar{b}}_{1}$	${\bar{b}}_{2}$	*k*_s_
1	0.2039	0.0707	2.4733	0.6868	−1.0032	−0.0319
2	0.0503	0.0152	0.3736	1.2695	−0.2733	3.8860
